# Determinants of Vaccine Acceptance against COVID-19 in China: Perspectives on Knowledge and DrVac-COVID19S Scale

**DOI:** 10.3390/ijerph182111192

**Published:** 2021-10-25

**Authors:** Chen Dong, Qian Liang, Tanao Ji, Jun Gu, Jian Feng, Min Shuai, Xiaoming Zhang, Rui Zhao, Zhifeng Gu

**Affiliations:** 1 Research Center of Clinical Medicine, Affiliated Hospital of Nantong University, Nantong 226001, China; 2013510019@stmail.ntu.cn (C.D.); 2013310135@stmail.ntu.cn (Q.L.); 2 Department of Nursing, Affiliated Hospital of Nantong University, Nantong 226001, China; minshuai@ntu.edu.cn; 3 Department of Respiratory, Affiliated Hospital of Nantong University, Nantong 226001, China; 2013300097@stmail.ntu.cn (T.J.); jungu@ntu.edu.cn (J.G.); jianfeng@ntu.edu.cn (J.F.); 4 Key Laboratory of Molecular Virology & Immunology, Institut Pasteur of Shanghai, Chinese Academy of Sciences, Shanghai 200025, China; xmzhang@ips.ac.cn; 5 Department of Rheumatology, Affiliated Hospital of Nantong University, Nantong 226001, China

**Keywords:** COVID-19, SARS-CoV-2, vaccine, acceptance, knowledge, attitude

## Abstract

**Background:** This study determined the knowledge and attitudes regarding COVID-19 and assessed the acceptance of the COVID-19 vaccine among the Chinese population. **Methods:** An online and offline cross-sectional study was conducted from 1 to 18 June 2021 among the Chinese population. Demographic characteristics, attitudes, knowledge, values, impact, and autonomy regarding the COVID-19 vaccine were collected using questionnaire. The variables in our study were analyzed by Mann-Whitney test and chi-square test. **Results:** A total of 93.8% participants were willing to be vaccinated, 2.7% refused, and 3.5% hesitated. In regards to knowledge about the COVID-19 vaccine, 94.3% citizens surveyed knew about the spread of droplets and 65% had knowledge about surfaces touched by an infected person. In addition, 93.8% of participants had knowledge of the common symptoms related to COVID-19, such as fever and cough (93.8%), shortness of breath/anorexia/fatigue/nausea/vomiting/diarrhea (80.2%), and panic and chest tightness (69.4%). Most participants had a strong self-prevention awareness, such as washing hands regularly (92.1%) and wearing a facemask (94.1%). Besides, over ninety percent of respondents would report exposure to SARS-CoV-2 (96.6%) and exposure to symptoms possibility related to COVID-19 (92.9%). If necessary, most respondents would agree to isolate at home (93.5%) or an isolation in hospital (96.3%). Knowledge of COVID-19, including transmission, symptoms, protective measures, and vaccines itself, is associated with vaccination attitude. Values, perceived impacts, knowledge, and autonomy, assessed by the scale of DrVac-COVID19S, have also been revealed as important determinants to vaccine acceptance. **Conclusions:** Almost 93% of Chinese people surveyed in this study showed a willing attitude toward COVID-19 vaccination. Based on the above results, government and social workers can take measures from these perspectives to improve the vaccination attitude, so as to increase vaccine immunization rates.

## 1. Introduction

The ongoing Coronavirus Disease 2019 (COVID-19) pandemic in 2019 has become a major global health threat, with more than 208 million cases and nearly 4 million deaths worldwide (data from the World Health Organization). COVID-19 is caused by the novel severe acute respiratory syndrome coronavirus 2 (SARS-CoV-2), which leads to series of symptoms, including fever, dry cough, short of breath and multiple organs and systems disfunction [1]. COVID-19 can cause not only high mortality and morbidity, but also serious effects on anxiety, depression, social distance, social burden, and economic development [2].

To date, there is no effective therapy for COVID-19. Therefore, the key management of COVID-19 pandemic is the availability of effective vaccines, which helps reduce transmission, hospitalization, and the need for intensive care [3]. It has been estimated that the basic reproductive number for SARS-CoV-2 was at 2.4–3.4, so that only large-scale, equitable access and distribution of a COVID-19 vaccine can achieve herd immunity against SARS-CoV-2, and uptake of vaccine should reach 60–72% [4]. However, successful vaccination programs are largely affected by acceptance rates, which are not satisfactory on a global scale [5]. A global survey showed that potential acceptance of the COVID-19 vaccine across countries and regions varies from 27.3% to 88.6% [6]. One of biggest barriers to full population inoculation against COVID-19 is vaccine hesitancy.

In 2015, the WHO Strategic Advisory Group of Experts on Immunization defined vaccine hesitancy as a “delay in acceptance or refusal of vaccination despite availability of vaccination service” [7]. The vaccine hesitancy of COVID-19 is increasing globally [8]. When a new vaccine is introduced, vaccine hesitancy occurs and is influenced by many factors [9,10]. These include the environmental factors, host factors, and agents factors, such as safety and effectiveness of vaccines, adverse health outcomes, misunderstandings about the necessity of vaccination, lack of trust in the health system, and lack of community understanding of vaccine-preventable diseases [11,12]. Therefore, studying the determinants of COVID-19 vaccine hesitancy can help in guiding interventional measures aimed at building and maintaining responses to enhance trust in, and acceptance of, the vaccine and those who deliver it.

In China, Hai Fang et al. reported that 91.3% of Chinese people would accept COVID-19 and 52.2% persons wanted to get vaccinated as quickly as possible after the vaccine became available in March 2020 [13]. In June 2020, acceptance rates of COVID-19 vaccines ranged up to 90% in China [6]. Moreover, the Chinese version Drivers of COVID-19 Vaccination Acceptance Scale (DrVac-COVID19S) has been used to assess the values, impacts, knowledge, and autonomy since March 2021, and the scale deeply enhances the interpretability of research results [14]. More importantly, China is a huge, diverse country, so more research about the acceptance and delineation of demographics related to COVID-19 vaccines in China would be helpful and can provide a theoretical basis for the global epidemic management. In the present study, we conducted a survey to assess the public attitude to COVID-19 vaccination and explore contributing factors of COVID-19 vaccination attitude, so as to provide a theoretical basis and intervention directions for healthcare providers and policymakers and improve the vaccination rate and finally achieve effective epidemic management.

## 2. Methodology

### 2.1. Participants

This cross-sectional study was conducted in China during June 2021. Firstly, participants were recruited online via snowball sampling (a type of convenience sampling) using the software of Wen Juan Xing (Changsha Ranxing Information Technology Co., Ltd., Changsha City, China). The software, similar to Qualtrics, SurveyMonkey, or CloudResearch, can provide online questionnaire design and survey functions and is the largest online survey platform in China allowing for an authentic, diverse, and representative sample. Secondly, in order to reduce selection bias of missing samples based on old age, lower income, rural, no internet, etc., an offline survey was also conducted in villages in Nantong City, Jiangsu Province, China. Based on the exclusion criteria of participants under the age of 18, the final sample consisted of 1910 individuals.

### 2.2. Self-Reported Questionnaires

Demographic variables were the following: gender, age, place of residence, marital status, education, income/year, use tobacco or alcohol, health status, live alone or not, have children in home or not.

A knowledge questionnaire regarding the spread, common symptoms, and protective measures of COVID-19 was designed referring to China’s national conditions and existing research, especially Abdelhafiz AS’s reports [15].

The Drivers of COVID-19 Vaccination Acceptance Scale (DrVac-COVID19S) was also conducted in this study, which is an instrument assessing attitudes and considerations in COVID-19 vaccines [14]. A higher score in DrVac-COVID19S indicates higher level of COVID-19 vaccine acceptance. The scale contains four dimensions of values, impacts, knowledge, and autonomy, revealing the underlying mechanism of different attitudes to COVID-19 vaccination.

### 2.3. Data Analysis

The variables in our study all involved abnormal distribution data, which were demonstrated in median (IQR) and analyzed by Mann-Whitney test. Descriptive statistics also involved frequencies (%) for categorical variables and group differences were assessed using the chi-square test. Statistical significance was considered when *p* < 0.05 (two-sided). All analyses were performed using SPSS version 21.0.

## 3. Results

### 3.1. Participant Characteristics

In this study, we recruited 1910 citizens in China and assessed their attitudes about COVID-19 vaccines, socioeconomic status, knowledge about COVID-19 (including spread ways, common symptoms, prevention measures), and knowledge about COVID-19 vaccines. In the present study, 93.8% participants were willing to be vaccinated, 2.7% refused, and 3.5% hesitated. Age, place of residence, marital status, educational level, income, and health status showed a significant association with acceptance. Overall, the majority of participants surveyed were positive about COVID-19 vaccination.

### 3.2. Differences between Willing/Unwilling/Hesitant Attitudes in Relation to Socioeconomic Items

**Figure 1:**
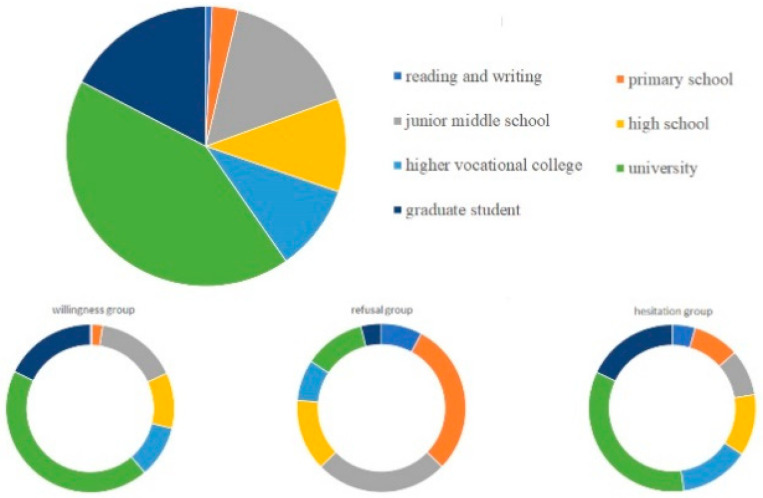
Pie charts of educational level in different attitude groups of COVID-19 vaccines.

Among all the participants, citizens aged over 50 years old showed higher refusal of vaccines when compared with young adults. In total, 118/1910 persons were skeptical of being vaccinated and nearly forty percent of people over 60-years-old refused or hesitated to get the vaccine. Participants with low incomes and chronic disease were more likely to refuse to be vaccinated. Interestingly, individuals with lower education were the largest group who refused to get vaccinated, while the biggest survey group hesitating to get vaccinated was actually the highly educated crowd. Participants with a primary school educational level were most likely to refuse vaccination, followed by individuals with junior middle school education. Moreover, participants with university educational experience were mostly likely to hesitate regarding COVID-19 vaccination and individuals with higher educational level of high school showed more possibility of hesitation (Figure 1). In addition, smokers or non-smokers and drinkers or non-drinkers showed no significant statistical differences in attitudes of willingness or refusal/hesitation, but the number of drinkers and smokers was relatively fewer than non-drinkers and non-smokers in this study, indicating the importance of relevant further study. (Table 1).

### 3.3. Differences between Willing/Unwilling/Hesitant Attitudes in Relation to Knowledge of COVID-19

Most participants were aware of the main ways SARS-CoV-2 spreads. As shown in Table 2, a total of 94.3% citizens knew about the spread of droplets from an infected person and 65% knew about surfaces touched by an infected person. Among the participants who refused to be vaccinated, 37.3% citizens did not know about the spread of droplets from infected people and 72.5% did not realize it could spread via surfaces touched by an infected person. Among participants who refused, 80.4% did not know about the transmission aerosols, while 88.2% of them did not know about transmission through food and water, same as oral–fecal transmission.

In addition, 93.8% participants had knowledge of common symptoms related to COVID-19, such as fever and cough (93.8%), shortness of breath/anorexia/fatigue/nausea/vomiting/diarrhea (80.2%), and panic and chest tightness (69.4%). Among the participants who were willing to be vaccinated, 95.2% knew the symptoms of fever and cough and 82.5% were aware of shortness of breath/anorexia/fatigue/nausea/vomiting/diarrhea, while 71.7% knew about panic and chest tightness and 67.7% knew about body aches. Nevertheless, many citizens lacked awareness of SARS-CoV-2 transmission, including via polluted food/water, oral–fecal transmission, and the conjunctival congestion of COVID-19 symptoms. All the above are shown in Table 2.

### 3.4. Differences between Willing/Unwilling/Hesitant Attitudes in Relation to Preventive Measures/Behaviors of COVID-19

Daily behaviors related to COVID-19 prevention also were assessed in this study (Table 3). Among Chinese citizens, 92.1% of them washed hands regularly and 94.1% of them usually wore a facemask. In addition, 96.6% of participants would report exposure to SARS-CoV-2 and 92.9% of participants would actively report the exposure of symptoms possibility related to COVID-19. If necessary, 93.5% persons would agree to isolate at home and 96.3% would agree to be isolated at an isolation hospital. A total of 91.8% of Chinese participants were concerned about the latest news about the spread of the COVID-19 in China, and 89.8% of Chinese people were concerned about the global epidemic. Meanwhile, 92.5% participants would get the knowledge and follow the instruction if it was available. It indicates that Chinese pay attention to the COVID-19 epidemic and are in a good self-management state, which have become the necessary factors for the country to achieve effective epidemic control. All the above are shown in Table 3 and Table S1.

### 3.5. Differences between Willing/Unwilling/Hesitant Attitudes in Relation to Knowledge of COVID-19 Vaccination

As shown in Table 4 and Table S2, the scale of DrVac-COVID19S was used in this study to assess the knowledge of vaccination. All the participants achieved the overall score of 68 (62.76) and the score of the willing group was to 69 (63.77). Participants with hesitant attitudes showed the lowest score of 55 (49.62), followed by the group who refused with 57 (49.63). The four dimensions of DrVac-COVID19S, including value, impacts, knowledge, and autonomy were all key traits for participants to decide to be vaccinated or not. The scores of the four dimensions were all higher among participants who will to be vaccinated than those who refused or hesitated. Moreover, each item of DrVac-COVID19S shows the statistical difference among three groups, including the items assessing vaccine efficacy, importance, autonomous choice, and mechanisms of the vaccine.

### 3.6. Logistic Regression Analysis of Vaccination Attitudes of Willingness and Hesitation (Refusal)

Next, we used logistic regression analysis to find the key factors that affect vaccine acceptance in China. Marital status and health status were predictors of vaccination attitude. Individuals who consciously wore a facemask or believed the protective effect of vaccines were positively associated with the likelihood of vaccine acceptance. All the above are shown in Table 5.

## 4. Discussion

Although the epidemic prevention policies of countries around the world are different, governments of all countries are actively pursuing vaccination projects. More than 100 vaccine development projects are being conducted and some vaccines have been put into use [16]. A major hindrance facing governments and health organizations is the low vaccination rate relative to requirements of herd immunity. In this study, almost 93% of Chinese participants surveyed reported intending to be vaccinated. The percentage is similar to previous studies in China [13,17] and higher than some studies from other country [18,19,20,21,22,23]. A study found relatively high acceptance rates (>80%) in Asian countries was related to citizens’ high trust in governments [6]. In Asia, the acceptance in Malaysia was 94.3%, 67.0–93.3% in Indonesia, and 79.8% in South Korea, which was relatively higher than most European and American countries [20,24]. Surprisingly, vaccinations are largely accepted in low- and middle-income countries [25]. For instance, about 73% of adult Egyptians (N = 559) would be willing to be vaccinated [15]. On the contrary, the results are quite different in some developed countries. Researchers in England reported 55.8% participants would accept a COVID-19 vaccine [26]. The acceptance of the vaccine in Italy was only 59%, while France was 58.9–62% during the pandemic [19,27]. It reported that 50–67% Americans refused and 25% said they will never get COVID-19 vaccines [28]. Therefore, it is necessary to identify factors related to vaccine acceptance and hesitancy to implement policy changes and help public health experts determine the conceptual framework and educational activities aimed at raising this awareness in the general population.

Different countries have different influencing factors for vaccine hesitancy. In England, 40.5% hesitated, related to factors of lower income, lack of belief in safety and effectiveness [26]. While in Egypt, the attitude to vaccines was associated with knowledge about COVID-19 [15]. Acceptance of the COVID-19 vaccine in Indonesia is influenced by the effectiveness of the vaccine [24]. It has been demonstrated that vaccine safety and effectiveness strongly influence vaccine acceptance in Australia [29]. In our studies, the results of DrVac-COVID19S suggest that individuals’ knowledge about COIVD-19 vaccines is closely related to their vaccination attitude. In China, most citizens comprehend the knowledge of COVID-19 and it was also an important factor for COVID-19 vaccination attitudes, including around transmission, symptoms, and preventive behavior. In the present study, we found that most Chinese had a strong sense of self-management to prevent COVID-19 (washing hands, wearing facemasks regularly, and active reporting, etc.) and more awareness of social participation (following the updates about the spread of virus at home and abroad, etc.). The sense and behavior of self-management was associated with vaccination intention, similarly to the report in Egypt [15]. As expected, individuals with higher educational levels had higher rate of vaccine acceptance, which might be related to their higher knowledge of the pandemic. However, individuals with high educational level also showed more possibility of COVID-19 vaccination hesitation and the phenomenon should be paid more attention. Conversely, older individuals were more skeptical to get vaccinated in our studies, due to them lacking knowledge and worrying about the greater risks of side effects. Although COVID-19 vaccination is free in China, lower income was still associated with vaccine hesitancy and possibly because of these participants have difficulty of accessing correct information about vaccines or the pandemic, and especially the indoctrination of error messages. Therefore, the government and public health experts must take necessary measures to provide more accessible knowledge and encourage positive attitudes to vaccination against COVID-19. An educational framework must also be developed for citizens to advertise the benefits of timely vaccination and actively disseminate accurate information about COVID-19. On other hand, logistic regression showed that the health status is also an important determinant of vaccination attitude. Unsurprisingly, participants with chronic disease status showed more concern on real or perceived risks associated with immunization [30] with a negative effect on vaccination attitude [31,32].

The study has some limitations. Firstly, the conclusions drawn by the cross-sectional survey method may not be so relevant; therefore, further longitudinal research is needed. Secondly, the design of online and offline survey protocol still risks potential selection biases. For example, people who were not accessible in this study are more likely to have a negative attitude towards COVID-19 vaccination. However, our results still confirm and expand results and conclusions from previous related studies. In this study, the types of chronic diseases and the vaccine acceptance of patients with certain diseases are not detailed and comprehensive, which need to be further studied.

## 5. Conclusions

The Chinese participants surveyed in this study had fuller understanding of COVID-19 and vaccines, and showed higher acceptance of vaccination. Knowledge of COVID-19, including transmission, symptoms, protective measures, and vaccines themselves, was associated with vaccination attitude. Values, perceived impacts, knowledge, and autonomy, assessed by the scale of DrVac-COVID19S, were also revealed as important determinants to vaccine acceptance. Based on this, government and social workers can take measures from these perspectives to improve the vaccination attitude, so as to increase vaccine immunization rates.

## Figures and Tables

**Table 1:** The differences between willing, unwilling, and hesitant attitudes in relation to socioeconomic items.

Items	All	Attitude of Being Vaccinated			*p*
		Willingness	Refusal	Hesitation	
Attitude of vaccination	1910 (100)	1792 (93.8)	51 (2.7)	67 (3.5)	---
Age, years					<0.001
18–<30	423 (22.1)	407 (22.7)	6 (11.8)	10 (14.9)	
30–<40	598 (31.3)	576 (32.1)	6 (11.8)	16 (23.9)	
40–<50	535 (28)	510 (28.5)	8 (15.7)	17 (25.4)	
50–<60	271 (14.2)	249 (13.9)	10 (19.6)	12 (17.9)	
≥60	83 (4.3)	50 (2.8)	21 (41.2)	12 (17.9)	
Gender					0.955
male	713 (37.3)	670 (37.4)	18 (35.3)	25 (37.3)	
female	1199 (62.7)	1122 (62.6)	33 (64.7)	42 (62.7)	
Place of residence					<0.001
urban	1503 (78.7)	1423 (79.4)	27 (52.9)	53 (79.1)	
Rural	407 (21.3)	369 (20.6)	24 (47.1)	14 (20.9)	
Marital status					0.026
married	1550 (81.2)	1444 (80.6)	48 (94.1)	58 (86.6)	
others	360 (18.8)	348 (19.4)	3 (5.9)	9 (13.4)	
Educational level					<0.001
reading and writing	14 (0.7)	7 (0.4)	4 (7.8)	3 (4.5)	
primary school	57 (3)	36 (2)	15 (29.4)	6 (9)	
junior middle school	301 (15.8)	282 (15.7)	13 (25.5)	6 (9)	
high school	206 (10.8)	191 (10.7)	7 (13.7)	8 (11.9)	
higher vocational college	192 (10)	179 (10)	4 (7.8)	9 (13.4)	
university	808 (42.3)	779 (43.5)	6 (11.8)	23 (34.3)	
graduate student	332 (17.4)	318 (17.7)	2 (3.9)	12 (17.9)	
Yearly per capita income, RMB					<0.001
<50,000	468 (24.5)	413 (23.1)	34 (66.7)	21 (31.3)	
50,000–100,000	658 (34.5)	624 (34.8)	12 (23.5)	22 (32.8)	
>100,000	784 (41)	755 (42.1)	5 (9.8)	24 (35.8)	
Tobacco use					0.940
yes	208 (10.9)	194 (10.8)	6 (11.8)	8 (11.9)	
no	1702 (89.1)	1598 (89.2)	45 (88.2)	59 (88.1)	
Alcohol use					0.532
yes	104 (5.4)	99 (5.5)	1 (2)	4 (6)	
no	1806 (94.6)	1693 (94.5)	50 (98)	63 (94)	
Health status					<0.001
health	1647 (86.2)	1596 (89.1)	11 (21.6)	40 (59.7)	
chronic disease	263 (13.8)	196 (10.9)	40 (78.4)	27 (40.3)	
Living alone					0.756
yes	126 (6.6)	120 (6.7)	3 (5.9)	3 (4.5)	
no	1784 (93.4)	1672 (93.3)	48 (94.1)	64 (95.5)	
Have children at home					0.324
yes	1573 (82.4)	1476 (82.4)	45 (88.2)	52 (77.6)	
no	337 (17.6)	316 (17.6)	6 (11.8)	15 (22.4)	

**Table 2:** The differences between willing, unwilling, and hesitant attitudes in relation to knowledge of COVID-19.

Items	All	Attitude of Being Vaccinated			*p*
		Willingness	Refusal	Hesitation	
**COVID-19 spreads by**					
1. Droplets of affected person (with cough or expiration)					<0.001
yes	1802 (94.3)	1711 (95.5)	32 (62.7)	59 (88.1)	
no	108 (5.7)	81 (4.5)	19 (37.3)	8 (11.9)	
2. Surfaces touched by affected person					<0.001
yes	1242 (65)	1193 (66.6)	14 (27.5)	35 (52.2)	
no	668 (35)	599 (33.4)	37 (72.5)	32 (47.8)	
3. Aerosol transmission					<0.001
yes	1150 (60.2)	1113 (62.1)	10 (19.6)	27 (40.3)	
no	760 (39.8)	679 (37.9)	41 (80.4)	40 (59.7)	
4.Transmission through food and water					<0.001
yes	858 (44.9)	828 (46.2)	6 (11.8)	24 (35.8)	
no	1052 (55.1)	964 (53.8)	45 (88.2)	43 (64.2)	
5. Oral–fecal transmission					<0.001
yes	832 (43.6)	806 (45.0)	6 (11.8)	20 (29.9)	
no	1078 (56.4)	986 (55.0)	45 (88.2)	47 (70.1)	
**Common symptoms include**					
1. Fever and cough					<0.001
yes	1792 (93.8)	1706 (95.2)	30 (58.8)	56 (83.6)	
no	118 (6.2)	86 (4.8)	21 (41.2)	11 (16.4)	
2. Shortness of breath, anorexia, fatigue, nausea, vomiting, diarrhea					<0.001
yes	1532 (80.2)	1478 (82.5)	12 (23.5)	42 (62.7)	
no	378 (19.8)	314 (17.5)	39 (76.5)	25 (37.3)	
3. Panic and chest tightness					<0.001
yes	1325 (69.4)	1284 (71.7)	7 (13.7)	34 (50.7)	
no	585 (30.6)	508 (28.3)	44 (86.3)	33 (49.3)	
4. Body aches					<0.001
yes	1247 (65.3)	1213 (67.7)	6 (11.8)	28 (41.8)	
no	663 (34.7)	579 (32.3)	45 (88.2)	39 (58.2)	
5. Conjunctival congestion					<0.001
yes	675 (35.3)	656 (36.6)	5 (9.8)	14 (20.9)	
no	1235 (64.7)	1136 (63.4)	46 (90.2)	53 (79.1)	

**Table 3:** The differences between willing, unwilling, and hesitant attitudes in relation to preventive measures of COVID-19.

Items	All	Attitude of Being Vaccinated			*p*
		Willingness	Refusal	Hesitation	
1. When I meet my friends and colleagues, I will always greet them with a handshake					0.017
agree	422 (22.1)	410 (22.9)	3 (5.9)	9 (13.4)	
uncertain	508 (26.6)	473 (26.4)	14 (27.5)	21 (31.3)	
disagree	980 (51.3)	909 (50.7)	34 (66.7)	37 (55.2)	
2. When I meet my friends and colleagues, I will always greet them with a hug					0.280
agree	181 (9.5)	176 (9.8)	2 (3.9)	3 (4.5)	
uncertain	414 (21.7)	390 (21.8)	12 (23.5)	12 (17.9)	
disagree	1315 (68.8)	1226 (68.4)	37 (72.5)	52 (77.6)	
3. I wash my hands regularly and for enough period of time					<0.001
agree	1760 (92.1)	1702 (95)	13 (25.5)	45 (67.2)	
uncertain	84 (4.4)	63 (3.5)	13 (25.5)	8 (11.9)	
disagree	66 (3.5)	27 (1.5)	25 (49)	14 (20.9)	
4. I usually put a facemask to protect myself from the risk of infection					<0.001
agree	1798 (94.1)	1695 (94.6)	40 (78.4)	63 (94)	
uncertain	88 (4.6)	79 (4.4)	5 (9.8)	4 (6)	
disagree	24 (1.3)	18 (1.0)	6 (11.8)	0 (0)	
5. If I find that I contacted a person infected with the virus, I will inform the health authorities					<0.001
agree	1846 (96.6)	1756 (98.0)	33 (64.7)	57 (85.1)	
uncertain	48 (2.5)	27 (1.5)	13 (25.5)	8 (11.9)	
disagree	16 (0.8)	9 (0.5)	5 (9.8)	2 (3)	
6. If I have any of the symptoms associated with the disease, I will inform the health authorities					<0.001
agree	1775 (92.9)	1691 (94.4)	31 (60.8)	53 (79.1)	
uncertain	108 (5.7)	81 (4.5)	15 (29.4)	12 (17.9)	
disagree	27 (1.4)	20 (1.1)	5 (9.8)	2 (3)	
7. If I find that I contacted a person infected with the virus, I agree to be isolated at home for a certain period of time until it is proven that I am free from the disease					<0.001
agree	1786 (93.5)	1686 (94.1)	41 (80.4)	59 (88.1)	
uncertain	51 (2.7)	41 (2.3)	4 (7.8)	6 (9)	
disagree	73 (3.8)	65 (3.6)	6 (11.8)	2 (3)	
8. If I found that I contacted a person infected with the virus, I agree to be isolated at an isolation hospital for a certain period of time until it is proven that I am free from the disease					<0.001
agree	1839 (96.3)	1742 (97.2)	41 (80.4)	56 (83.6)	
uncertain	55 (2.9)	40 (2.2)	5 (9.8)	10 (14.9)	
disagree	16 (0.8)	10 (0.6)	5 (9.8)	1 (1.5)	
9. If I am asked to be isolated for a certain period of time, I think my salary will continue during this period					0.171
agree	1139 (59.6)	1071 (59.8)	30 (58.8)	38 (56.7)	
uncertain	429 (22.5)	409 (22.8)	7 (13.7)	13 (19.4)	
disagree	342 (17.9)	312 (17.4)	14 (27.5)	16 (23.9)	
10. If I am asked to be isolated for a certain period of time, my salary should be continued during this period					0.311
agree	1389 (72.8)	1299 (72.5)	43 (84.3)	47 (70.1)	
uncertain	339 (17.7)	319 (17.8)	7 (13.7)	13 (19.4)	
disagree	182 (9.5)	174 (9.7)	1 (2)	7 (10.4)	
11. If there is an available lab test for detection of the virus, I am willing to do it					<0.001
agree	1865 (97.6)	1756 (98)	48 (94.1)	61 (91)	
uncertain	40 (2.1)	31 (1.7)	3 (5.9)	6 (9)	
disagree	5 (0.3)	5 (0.3)	0 (0)	0 (0)	
12. If there is an available vaccine for the virus, I am willing to get it					<0.001
agree	1773 (92.8)	1740 (97.1)	10 (19.6)	23 (34.3)	
uncertain	76 (4)	43 (2.4)	4 (7.8)	29 (43.3)	
disagree	61 (3.2)	9 (0.5)	37 (72.5)	15 (22.4)	
13. I usually follow the updates about the spread of the virus in my country					<0.001
agree	1753 (91.8)	1693 (94.5)	16 (31.4)	44 (65.7)	
uncertain	116 (6.1)	81 (4.5)	20 (39.2)	15 (22.4)	
disagree	41 (2.1)	18 (1)	15 (29.4)	8 (11.9)	
14. I usually follow the updates about the spread of the virus worldwide					<0.001
agree	1715 (89.8)	1658 (92.5)	14 (27.5)	43 (64.2)	
uncertain	149 (7.8)	110 (6.1)	22 (43.1)	17 (25.4)	
disagree	46 (2.4)	24 (1.3)	15 (29.4)	7 (10.4)	
15. If a lecture about the virus is organized near me, I will attend it					<0.001
agree	1317 (69)	1280 (71.4)	10 (19.6)	27 (40.3)	
uncertain	526 (27.5)	459 (25.6)	32 (62.7)	35 (52.2)	
disagree	67 (3.5)	53 (3)	9 (17.6)	5 (7.5)	
16. If flyers or brochures that include information about the disease are distributed, I will read them and follow the instructions mentioned in them					<0.001
agree	1767 (92.5)	1693 (94.5)	23 (45.1)	51 (76.1)	
uncertain	117 (6.1)	82 (4.6)	21 (41.2)	14 (20.9)	
disagree	26 (1.4)	17 (0.9)	7 (13.7)	2 (3)	

**Table 4:** The differences between willing, unwilling, and hesitant attitudes in relation to knowledge of COVID-19 vaccination.

Items	All	Attitude of Being Vaccinated			*p*
		Willingness	Refusal	Hesitation	
Total score of DrVac-COVID19S	68 (62.76)	69 (63.77)	57 (49.63)	55 (49.62)	<0.001
value	18 (17.21)	19 (18.21)	16 (12.18)	15 (13.18)	<0.001
impacts	16 (14.19)	17 (14.19)	15 (12.18)	14 (12.17)	<0.001
knowledge	16 (14.19)	16 (14.19)	9 (7.11)	11 (9.13)	<0.001
autonomy traits	18 (15.20)	18 (15.20)	18 (16.18)	14 (12.17)	<0.001
1. Vaccination is a very effective way to protect me against COVID-19.					<0.001
agree	1494 (78.2)	1430 (79.8)	30 (58.8)	34 (50.7)	
uncertain	305 (16)	261 (14.6)	18 (35.3)	26 (38.8)	
disagree	111 (5.8)	101 (5.6)	3 (5.9)	7 (10.4)	
2. I know very well how vaccination protects me from COVID-19.					<0.001
agree	1401 (73.4)	1378 (76.9)	6 (11.8)	17 (25.4)	
uncertain	337 (17.6)	310 (17.3)	4 (7.8)	23 (34.3)	
disagree	172 (9)	104 (5.8)	41 (80.4)	27 (40.3)	
3. It is important that I get the COVID-19 jab.					<0.001
agree	1811 (94.8)	1726 (96.3)	36 (70.6)	49 (73.1)	
uncertain	75 (3.9)	49 (2.7)	13 (25.5)	13 (19.4)	
disagree	24 (1.3)	17 (0.9)	2 (3.9)	5 (7.5)	
4. Vaccination greatly reduces my risk of catching COVID-19.					<0.001
agree	1757 (92)	1675 (93.5)	35 (68.6)	47 (70.1)	
uncertain	128 (6.7)	100 (5.6)	14 (27.5)	14 (20.9)	
disagree	25 (1.3)	17 (0.9)	2 (3.9)	6 (9)	
5. I understand how the flu jab helps my body fight the COVID-19 virus.					<0.001
agree	1534 (80.3)	1465 (81.8)	30 (58.8)	39 (58.2)	
uncertain	315 (16.5)	280 (15.6)	15 (29.4)	20 (29.9)	
disagree	61 (3.2)	47 (2.6)	6 (11.8)	8 (11.9)	
6. The COVID-19 jab plays an important role in protecting my life and that of others.					<0.001
agree	1727 (90.4)	1649 (92)	33 (64.7)	45 (67.2)	
uncertain	147 (7.7)	112 (6.2)	16 (31.4)	19 (28.4)	
disagree	36 (1.9)	31 (1.7)	2 (3.9)	3 (4.5)	
7. I feel under pressure to get the COVID-19 jab.					<0.001
agree	267 (14)	243 (13.6)	6 (11.8)	18 (26.9)	
uncertain	206 (10.8)	183 (10.2)	6 (11.8)	17 (25.4)	
disagree	1437 (75.2)	1366 (76.2)	39 (76.5)	32 (47.8)	
8. The contribution of the COVID-19 jab to my health and well-being is very important.					<0.001
agree	1669 (87.4)	1603 (89.5)	29 (56.9)	37 (55.2)	
uncertain	201 (10.5)	156 (8.7)	20 (39.2)	25 (37.3)	
disagree	40 (2.1)	33 (1.8)	2 (3.9)	5 (7.5)	
9. I can choose whether to get a COVID-19 jab or not.					<0.001
agree	1742 (91.2)	1647 (91.9)	46 (90.2)	49 (73.1)	
uncertain	68 (3.6)	52 (2.9)	4 (7.8)	12 (17.9)	
disagree	100 (5.2)	93 (5.2)	1 (2)	6 (9)	
10. How the COVID-19 jab works to protect my health is a mystery to me.					<0.001
agree	464 (24.3)	383 (21.4)	44 (86.3)	37 (55.2)	
uncertain	371 (19.4)	344 (19.2)	6 (11.8)	21 (31.3)	
disagree	1075 (56.3)	1065 (59.4)	1 (2)	9 (13.4)	
11. I get the COVID-19 jab only because I am required to do so.					<0.001
agree	262 (13.7)	242 (13.5)	2 (3.9)	18 (26.9)	
uncertain	139 (7.3)	118 (6.6)	8 (15.7)	13 (19.4)	
disagree	1509 (79)	1432 (79.9)	41 (80.4)	36 (53.7)	
12. Getting the COVID-19 jab has a positive influence on my health.					<0.001
agree	1113 (58.3)	1055 (58.9)	24 (47.1)	34 (50.7)	
uncertain	317 (16.6)	266 (14.8)	24 (47.1)	27 (40.3)	
disagree	480 (25.1)	471 (26.3)	3 (5.9)	6 (9)	

**Table 5:** Logistic regression analysis of vaccinated attitudes of willingness and hesitation (refusal).

	B	S.E.	*p*	OR	95% CI	
Place of residence	−0.576	0.398	0.147	0.562	0.258	1.226
Marital status	−1.023	0.499	0.040 *	0.360	0.135	0.956
Educational level	0.096	0.130	0.461	1.100	0.853	1.418
Yearly per capita income	0.075	0.209	0.718	1.078	0.716	1.623
Health status	−1.026	0.422	0.015 *	0.359	0.157	0.820
Item 1-knowledge of COVID-19 spreads	0.142	0.596	0.812	1.152	0.358	3.705
Item 2-knowledge of COVID-19 spreads	0.412	0.399	0.302	1.510	0.690	3.302
Item 3-knowledge of COVID-19 spreads	−0.374	0.413	0.365	0.688	0.306	1.545
Item 4-knowledge of COVID-19 spreads	0.504	0.411	0.220	1.655	0.740	3.703
Item 5-knowledge of COVID-19 spreads	−0.210	0.427	0.623	0.811	0.351	1.871
Item 1-knowledge of COVID-19 symptoms	−1.800	0.532	0.001 *	0.165	0.058	0.469
Item 2-knowledge of COVID-19 symptoms	0.043	0.462	0.925	1.044	0.422	2.581
Item 3-knowledge of COVID-19 symptoms	0.490	0.455	0.282	1.632	0.668	3.983
Item 4-knowledge of COVID-19 symptoms	−0.786	0.445	0.077	0.455	0.190	1.090
Item 5-knowledge of COVID-19 symptoms	−0.112	0.430	0.794	0.894	0.385	2.076
Item 1-knowledge of COVID-19 vaccination	0.339	0.265	0.201	1.404	0.835	2.360
Item 2-knowledge of COVID-19 vaccination	0.329	0.281	0.242	1.390	0.801	2.413
Item 3-knowledge of COVID-19 vaccination	0.664	0.459	0.148	1.943	0.791	4.776
Item 4-knowledge of COVID-19 vaccination	0.304	0.432	0.482	1.355	0.581	3.160
Item 5-knowledge of COVID-19 vaccination	0.304	0.275	0.268	1.355	0.791	2.321
Item 6-knowledge of COVID-19 vaccination	−1.044	0.481	0.030 *	0.352	0.137	0.903
Item 7-knowledge of COVID-19 vaccination	−0.348	0.201	0.083	0.706	0.477	1.046
Item 8-knowledge of COVID-19 vaccination	0.602	0.373	0.107	1.826	0.879	3.795
Item 9-knowledge of COVID-19 vaccination	−0.381	0.285	0.181	0.683	0.391	1.194
Item 10-knowledge of COVID-19 vaccination	−0.240	0.236	0.310	0.787	0.495	1.250
Item 11-knowledge of COVID-19 vaccination	−0.410	0.204	0.045 *	0.664	0.445	0.990
Item 12-knowledge of COVID-19 vaccination	−0.208	0.209	0.320	0.812	0.539	1.224
Item 1-preventive measure of COVID-19	0.068	0.193	0.724	1.070	0.734	1.562
Item 3-preventive measure of COVID-19	0.695	0.394	0.078	2.003	0.925	4.339
Item 4-preventive measure of COVID-19	−1.543	0.543	0.004 *	0.214	0.074	0.619
Item 5-preventive measure of COVID-19	1.134	0.767	0.139	3.109	0.692	13.973
Item 6-preventive measure of COVID-19	−0.875	0.702	0.213	0.417	0.105	1.651
Item 7-preventive measure of COVID-19	−0.227	0.571	0.691	0.797	0.260	2.438
Item 8-preventive measure of COVID-19	−0.074	0.798	0.926	0.928	0.194	4.434
Item 11-preventive measure of COVID-19	−0.873	0.617	0.157	0.418	0.125	1.400
Item 12-preventive measure of COVID-19	3.056	0.345	<0.001 *	21.237	10.794	41.785
Item 13-preventive measure of COVID-19	0.527	0.811	0.515	1.694	0.346	8.297
Item 14-preventive measure of COVID-19	−0.962	0.816	0.238	0.382	0.077	1.892
Item 15-preventive measure of COVID-19	−0.481	0.339	0.156	0.618	0.318	1.201
Item 16-preventive measure of COVID-19	−0.102	0.493	0.836	0.903	0.344	2.371

Notes: *: *p* < 0.05. Questions of significant items: Item 1-knowledge of COVID-19 symptoms: Fever and cough; Item 6-knowledge of COVID-19 vaccination: The COVID-19 jab plays an important role in protecting my life and that of others; Item 11-knowledge of COVID-19 vaccination: I get the COVID-19 jab only because I am required to do so; Item 4-preventive measure of COVID-19: I usually put a facemask to protect myself from the risk of infection; Item 12-preventive measure of COVID-19: If there is an available vaccine for the virus, I am willing to get it.

## Data Availability

The data presented in this study are available on request from the corresponding author. The data are not publicly available due to further exploration.

## Supplementary Materials

The following are available online at https://www.mdpi.com/article/10.3390/ijerph182111192/s1, Table S1: The differences between willing, refused, hesitated attitude among preventive measure of COVID-19, Table S2: The differences between willing, refused, hesitated attitude among knowledge of COVID-19 vaccination.
