# Supplementary material for: Determinants of Vaccine Acceptance against COVID-19 in China: Perspectives on Knowledge and DrVac-COVID19S Scale
Source: Int J Environ Res Public Health. 2021 Oct 25;18(21):11192. doi: 10.3390/ijerph182111192 (PMC8582645; doi:10.3390/ijerph182111192)
Supplement: Supplementary file 1 [file ijerph-18-11192-s001.zip › ijerph-1390428-supplementary.pdf]

Table S1. The differences between willing, refused, hesitated attitude among preventive measure of COVID-19.

**Items**	**All**	**Attitude of Being Vaccinated**			***P***
		**Willingness**	**Refusal**	**Hesitation**	
1. When I meet my friends and colleagues, I will always greet them with a handshake					0.001
strongly agree	106(5.5)	106(5.9)	0(0)	0(0)	
agree	316(16.5)	304(17)	3(5.9)	9(13.4)	
uncertain	508(26.6)	473(26.4)	14(27.5)	21(31.3)	
disagree	736(38.5)	672(37.5)	32(62.7)	32(47.8)	
strongly disagree	244(12.8)	237(13.2)	2(3.9)	5(7.5)	
2. When I meet my friends and colleagues, I will always greet them with a hug					0.012
strongly agree	68(3.6)	68(3.8)	0(0)	0(0)	
agree	113(5.9)	108(6)	2(3.9)	3(4.5)	
uncertain	414(21.7)	390(21.8)	12(23.5)	12(17.9)	
disagree	943(49.4)	866(48.3)	35(68.6)	42(62.7)	
strongly disagree	372(19.5)	360(20.1)	2(3.9)	10(14.9)	
3. I wash my hands regularly and for enough period of time					<0.001
strongly agree	907(47.5)	887(49.5)	4(7.8)	16(23.9)	
agree	853(44.7)	815(45.5)	9(17.6)	29(43.3)	
uncertain	84(4.4)	63(3.5)	13(25.5)	8(11.9)	
disagree	60(3.1)	22(1.2)	24(47.1)	14(20.9)	
strongly disagree	6(0.3)	5(0.3)	1(2)	0(0)	
4. I usually put a facemask to protect myself from the risk of infection					<0.001
strongly agree	996(52.1)	958(53.5)	17(33.3)	21(31.3)	
agree	802(42)	737(41.1)	23(45.1)	42(62.7)	
uncertain	88(4.6)	79(4.4)	5(9.8)	4(6)	
disagree	17(0.9)	11(0.6)	6(11.8)	0(0)	

strongly disagree	7(0.4)	7(0.4)	0(0)	0(0)	
5. If I find that I contacted a person infected with the virus, I will inform the health authorities					<0.001
strongly agree	1253(65.6)	1221(68.1)	5(9.8)	27(40.3)	
agree	593(31)	535(29.9)	28(54.9)	30(44.8)	
uncertain	48(2.5)	27(1.5)	13(25.5)	8(11.9)	
disagree	10(0.5)	3(0.2)	5(9.8)	2(3)	
strongly disagree	6(0.3)	6(0.3)	0(0)	0(0)	
6. If I have any of the symptoms associated with the disease, I will inform the health authorities					<0.001
strongly agree	1091(57.1)	1064(59.4)	5(9.8)	22(32.8)	
agree	684(35.8)	627(35)	26(51)	31(46.3)	
uncertain	108(5.7)	81(4.5)	15(29.4)	12(17.9)	
disagree	18(0.9)	12(0.7)	5(9.8)	1(1.5)	
strongly disagree	9(0.5)	8(0.4)	0(0)	1(1.5)	
7. If I find that I contacted a person infected with the virus, I agree to be isolated at home for a certain period of time until it is proven that I am free from the disease					<0.001
strongly agree	1184(62)	1144(63.8)	11(21.6)	29(43.3)	
agree	602(31.5)	542(30.2)	30(58.8)	30(44.8)	
uncertain	51(2.7)	41(2.3)	4(7.8)	6(9)	
disagree	43(2.3)	35(2)	6(11.8)	2(3)	
strongly disagree	30(1.6)	30(1.7)	0(0)	0(0)	
8. If I found that I contacted a person infected with the virus, I agree to be isolated at an isolation hospital for a certain period of time until it is proven that I am free from the disease					<0.001
strongly agree	1214(63.6)	1176(65.6)	11(21.6)	27(40.3)	
agree	625(32.7)	566(31.6)	30(58.8)	29(43.3)	
uncertain	55(2.9)	40(2.2)	5(9.8)	10(14.9)	
disagree	13(0.7)	7(0.4)	5(9.8)	1(1.5)	

strongly disagree	3(0.2)	3(0.2)	0(0)	0(0)	
9. If I am asked to be isolated for a certain period of time, I think my salary will continued during this period					<0.001
strongly agree	550(28.8)	540(30.1)	0(0)	10(14.9)	
agree	589(30.8)	531(29.6)	30(58.8)	28(41.8)	
uncertain	429(22.5)	409(22.8)	7(13.7)	13(19.4)	
disagree	259(13.6)	232(12.9)	14(27.5)	13(19.4)	
strongly disagree	83(4.3)	80(4.5)	0(0)	3(4.5)	
10. If I am asked to be isolated for a certain period of time, my salary should be continued during this period					<0.001
strongly agree	644(33.7)	632(35.3)	0(0)	12(17.9)	
agree	745(39)	667(37.2)	43(84.3)	35(52.2)	
uncertain	339(17.7)	319(17.8)	7(13.7)	13(19.4)	
disagree	133(7)	129(7.2)	1(2)	3(4.5)	
strongly disagree	49(2.6)	45(2.5)	0(0)	4(6)	
11. If there is an available lab test for detection of the virus, I am willing to do it					<0.001
strongly agree	1100(57.6)	1028(57.4)	38(74.5)	34(50.7)	
agree	765(40.1)	728(40.6)	10(19.6)	27(40.3)	
uncertain	40(2.1)	31(1.7)	3(5.9)	6(9)	
disagree	2(0.1)	2(0.1)	0(0)	0(0)	
strongly disagree	3(0.2)	3(0.2)	0(0)	0(0)	
12. If there is an available vaccine for the virus, I am willing to get it					<0.001
strongly agree	1001(52.4)	993(55.4)	3(5.9)	5(7.5)	
agree	772(40.4)	747(41.7)	7(13.7)	18(26.9)	
uncertain	76(4)	43(2.4)	4(7.8)	29(43.3)	
disagree	48(2.5)	4(0.2)	30(58.8)	14(20.9)	

strongly disagree	13(0.7)	5(0.3)	7(13.7)	1(1.5)	
13. I usually follow the updates about the spread of the virus in my country					<0.001
strongly agree	908(47.5)	892(49.8)	3(5.9)	13(19.4)	
agree	845(44.2)	801(44.7)	13(25.5)	31(46.3)	
uncertain	116(6.1)	81(4.5)	20(39.2)	15(22.4)	
disagree	30(1.6)	13(0.7)	11(21.6)	6(9)	
strongly disagree	11(0.6)	5(0.3)	4(7.8)	2(3)	
14. I usually follow the updates about the spread of the virus worldwide					<0.001
strongly agree	861(45.1)	844(47.1)	3(5.9)	14(20.9)	
agree	854(44.7)	814(45.4)	11(21.6)	29(43.3)	
uncertain	149(7.8)	110(6.1)	22(43.1)	17(25.4)	
disagree	34(1.8)	17(0.9)	11(21.6)	6(9)	
strongly disagree	12(0.6)	7(0.4)	4(7.8)	1(1.5)	
15. If a lecture about the virus is organized near me, I will attend it					<0.001
strongly agree	609(31.9)	602(33.6)	0(0)	7(10.4)	
agree	708(37.1)	678(37.8)	10(19.6)	20(29.9)	
uncertain	526(27.5)	459(25.6)	32(62.7)	35(52.2)	
disagree	60(3.1)	46(2.6)	9(17.6)	5(7.5)	
strongly disagree	7(0.4)	7(0.4)	0(0)	0(0)	
16. If flyers or brochures that include information about the disease are distributed, I will read them and follow the instructions mentioned in them					<0.001
strongly agree	865(45.3)	846(47.2)	8(15.7)	11(16.4)	
agree	902(47.2)	847(47.3)	15(29.4)	40(59.7)	
uncertain	117(6.1)	82(4.6)	21(41.2)	14(20.9)	
disagree	20(1)	11(0.6)	7(13.7)	2(3)	
strongly disagree	6(0.3)	6(0.3)	0(0)	0(0)	

Table S2. The differences between willing, refused, hesitated attitude among knowledge of COVID-19 vaccination.

Items	All	Attitude of Being Vaccinated			*P*
		Willingness	Refusal	Hesitation	
1. Vaccination is a very effective way to protect me against COVID-19.					<0.001
Strongly disagree	32(1.7)	32(1.8)	0(0)	0(0)	
Disagree	13(0.7)	10(0.6)	1(2)	3(3)	
Slightly disagree	66(3.5)	59(3.3)	2(5.9)	5(7.5)	
Neither disagree nor agree	305(16)	261(14.6)	18(35.3)	26(38.8)	
Slightly agree	236(12.4)	220(12.3)	5(9.8)	11(16.4)	
Agree	732(38.3)	691(38.6)	23(45.1)	18(26.9)	
Strongly agree	526(27.5)	519(29)	2(3.9)	5(7.5)	
2. I know very well how vaccination protects me from COVID-19.					<0.001
Strongly disagree	90(4.7)	44(2.5)	29(56.9)	17(25.4)	
Disagree	43(2.3)	24(1.3)	12(23.5)	7(10.4)	
Slightly disagree	39(2)	36(2)	0(0)	3(4.5)	
Neither disagree nor agree	337(17.6)	310(17.3)	4(7.8)	23(34.3)	
Slightly agree	223(11.7)	213(11.9)	1(2)	9(13.4)	
Agree	691(36.2)	685(38.2)	2(5.9)	3(4.5)	
Strongly agree	487(25.5)	480(26.8)	2(3.9)	5(7.5)	
3. It is important that I get the COVID-19 jab.					<0.001
Strongly disagree	8(0.4)	8(0.4)	0(0)	0(0)	
Disagree	6(0.3)	4(0.2)	2(3.9)	0(0)	
Slightly disagree	10(0.5)	5(0.3)	0(0)	5(7.5)	
Neither disagree nor agree	75(3.9)	49(2.7)	13(25.5)	13(19.4)	
Slightly agree	127(6.6)	103(5.7)	7(13.7)	17(25.4)	
Agree	812(42.5)	772(43.1)	20(39.2)	20(29.9)	

Strongly agree	872(45.7)	851(47.5)	9(17.6)	12(17.9)	
4. Vaccination greatly reduces my risk of catching COVID-19.					<0.001
Strongly disagree	11(0.6)	11(0.6)	0(0)	0(0)	
Disagree	4(0.2)	3(0.2)	0(0)	1(1.5)	
Slightly disagree	10(0.5)	3(0.2)	2(3.9)	5(7.5)	
Neither disagree nor agree	128(6.7)	100(5.6)	14(27.5)	14(20.9)	
Slightly agree	148(7.7)	126(7)	5(9.8)	17(25.4)	
Agree	747(39.1)	715(39.9)	14(27.5)	18(26.9)	
Strongly agree	862(45.1)	834(46.5)	16(31.4)	12(17.9)	
5. I understand how the flu jab helps my body fight the COVID-19 virus.					<0.001
Strongly disagree	18(0.9)	14(0.8)	3(5.9)	1(1.5)	
Disagree	13(0.7)	9(0.5)	0(0)	4(6)	
Slightly disagree	30(1.6)	24(1.3)	3(5.9)	3(4.5)	
Neither disagree nor agree	315(16.5)	280(15.6)	15(29.4)	20(29.9)	
Slightly agree	242(12.7)	226(12.6)	4(7.8)	12(17.9)	
Agree	683(35.8)	653(36.4)	14(27.5)	16(23.9)	
Strongly agree	609(31.9)	586(32.7)	12(23.5)	11(16.4)	
6. The COVID-19 jab plays an important role in protecting my life and that of others.					<0.001
Strongly disagree	10(0.5)	10(0.6)	0(0)	0(0)	
Disagree	7(0.4)	7(0.4)	0(0)	0(0)	
Slightly disagree	19(1)	14(0.8)	2(3.9)	3(4.5)	
Neither disagree nor agree	147(7.7)	112(6.3)	16(31.4)	19(28.4)	
Slightly agree	167(8.7)	141(7.9)	11(21.6)	15(22.4)	
Agree	802(42)	758(42.3)	20(39.2)	24(35.8)	
Strongly agree	758(39.7)	750(41.9)	2(3.9)	6(9)	
7. I feel under pressure to get the COVID-19 jab.					<0.001

Strongly disagree	668(35)	659(36.8)	3(5.9)	6(9)	
Disagree	594(31.1)	541(30.2)	34(66.7)	19(28.4)	
Slightly disagree	175(9.2)	166(9.3)	2(3.9)	7(10.4)	
Neither disagree nor agree	206(10.8)	183(10.2)	6(11.8)	17(25.4)	
Slightly agree	163(8.5)	145(8.1)	3(5.9)	15(22.4)	
Agree	57(3)	51(2.8)	3(5.9)	3(4.5)	
Strongly agree	47(2.5)	47(2.6)	0(0)	0(0)	
8. The contribution of the COVID-19 jab to my health and well-being is very important.					<0.001
Strongly disagree	10(0.5)	9(0.5)	0(0)	1(1.5)	
Disagree	8(0.4)	7(0.4)	1(2)	0(0)	
Slightly disagree	22(1.2)	17(0.9)	1(2)	4(6)	
Neither disagree nor agree	201(10.5)	156(8.7)	20(39.2)	25(37.3)	
Slightly agree	224(11.7)	197(11)	12(23.5)	15(22.4)	
Agree	768(40.2)	733(40.9)	16(31.4)	19(28.4)	
Strongly agree	677(35.4)	673(37.6)	1(2)	3(4.5)	
9. I can choose whether to get a COVID-19 jab or not.					<0.001
Strongly disagree	20(1)	19(1.1)	0(0)	1(1.5)	
Disagree	28(1.5)	28(1.6)	0(0)	0(0)	
Slightly disagree	52(2.7)	46(2.6)	1(2)	5(7.5)	
Neither disagree nor agree	68(3.6)	52(2.9)	4(7.8)	12(17.9)	
Slightly agree	126(6.6)	105(5.9)	9(17.6)	12(17.9)	
Agree	747(39.1)	681(38)	35(68.6)	31(46.3)	
Strongly agree	869(45.5)	861(48)	2(3.9)	6(9)	
10. How the COVID-19 jab works to protect my health is a mystery to me.					<0.001
Strongly disagree	461(24.1)	459(25.6)	0(0)	2(3)	

Disagree	430(22.5)	425(23.7)	1(2)	4(6)	
Slightly disagree	184(9.6)	181(10.1)	0(0)	3(4.5)	
Neither disagree nor agree	371(19.4)	344(19.2)	6(11.8)	21(31.3)	
Slightly agree	181(9.5)	170(9.5)	1(2)	10(14.9)	
Agree	177(9.3)	134(7.5)	28(54.9)	15(22.4)	
Strongly agree	106(5.5)	79(4.4)	15(29.4)	12(17.9)	
11. I get the COVID-19 jab only because I am required to do so.					<0.001
Strongly disagree	736(38.5)	720(40.2)	9(17.6)	7(10.4)	
Disagree	563(29.5)	514(28.7)	29(56.9)	20(29.9)	
Slightly disagree	210(11)	198(11)	3(5.9)	9(13.4)	
Neither disagree nor agree	139(7.3)	118(6.6)	8(15.7)	13(19.4)	
Slightly agree	145(7.6)	132(7.4)	1(2)	12(17.9)	
Agree	83(4.3)	77(4.3)	1(2)	5(7.5)	
Strongly agree	34(1.8)	33(1.8)	0(0)	1(1.5)	
12. Getting the COVID-19 jab has a positive influence on my health.					<0.001
Strongly disagree	252(13.2)	249(13.9)	1(2)	2(3)	
Disagree	182(9.5)	180(10)	1(2)	1(1.5)	
Slightly disagree	46(2.4)	42(2.3)	1(2)	3(4.5)	
Neither disagree nor agree	317(16.6)	266(14.8)	24(47.1)	27(40.3)	
Slightly agree	151(7.9)	126(7)	8(15.7)	17(25.4)	
Agree	534(28)	503(28.1)	16(31.4)	15(22.4)	
Strongly agree	428(22.4)	426(23.8)	0(0)	2(3)	
